# Supplementary material for: Transcriptome and Metabolome Analyses of Leaves from Cutting Rejuvenation of Ancient Cinnamomum camphora
Source: Int J Mol Sci. 2024 Jul 12;25(14):7664. doi: 10.3390/ijms25147664 (PMC11277138; doi:10.3390/ijms25147664)
Supplement: Supplementary file 1 [file ijms-25-07664-s001.zip › Supplementary Materials-Figure.pdf]

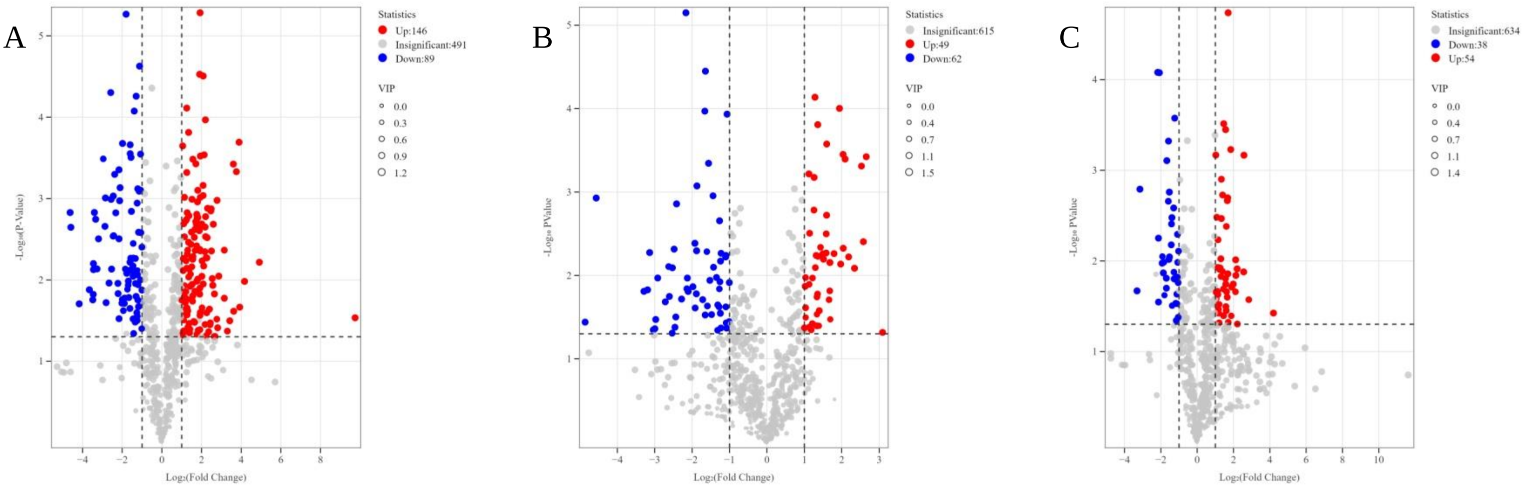

Figure S1. Volcano plots of DAMs (A: ML vs. RL; B: YL vs. RL; C: ML vs. YL).

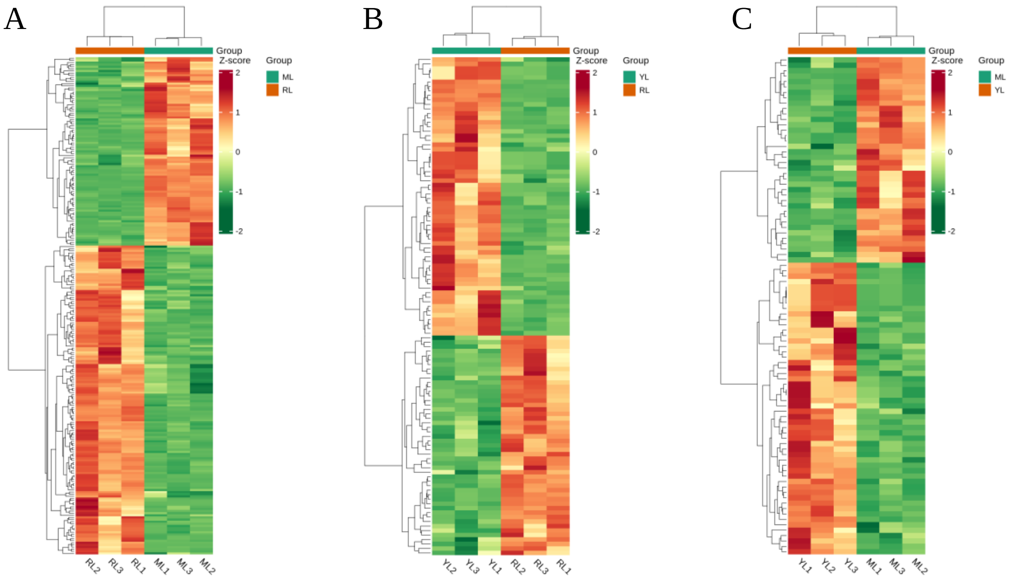

Figure S2. Heat map of cluster analyses of DAMs (A: ML vs. RL; B: YL vs. RL; C: ML vs. YL).

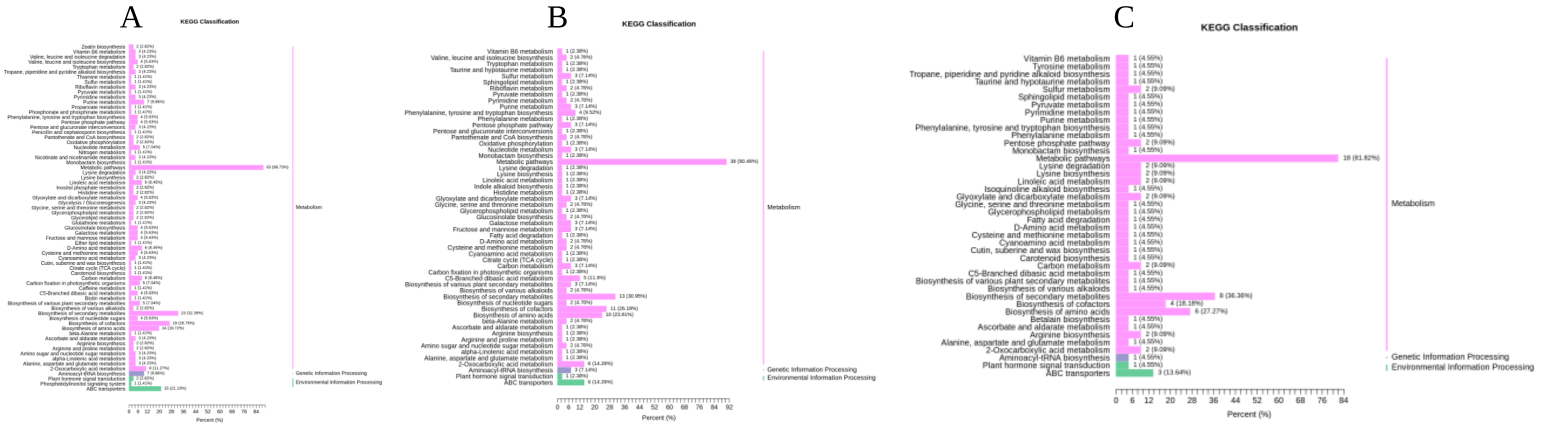

Figure S3. KEGG classification map of DAMs (A: ML vs. RL; B: YL vs. RL; C: ML vs. YL).

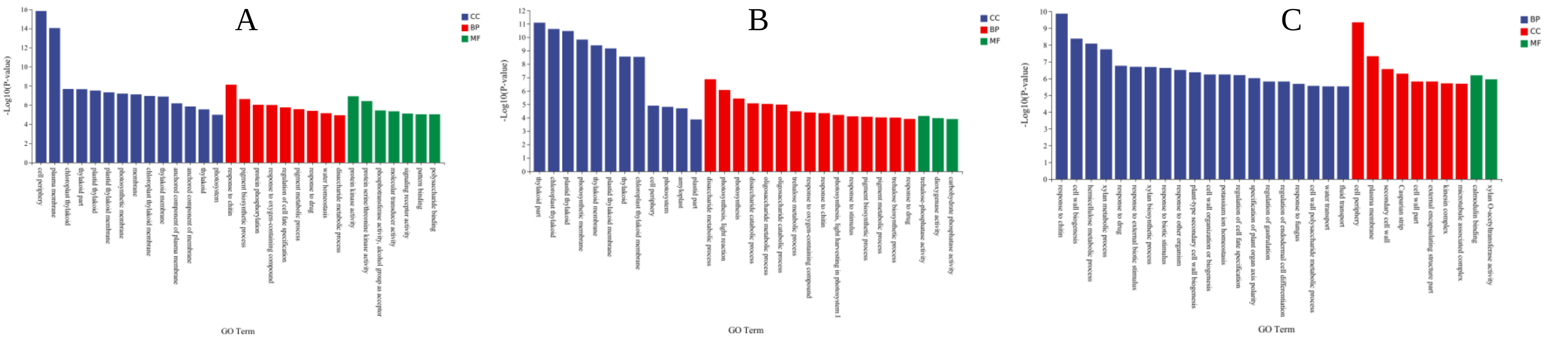

Figure S4. GO enrichment histogram of DEGs (A: ML vs. RL; B: YL vs. RL; C: ML vs. YL).

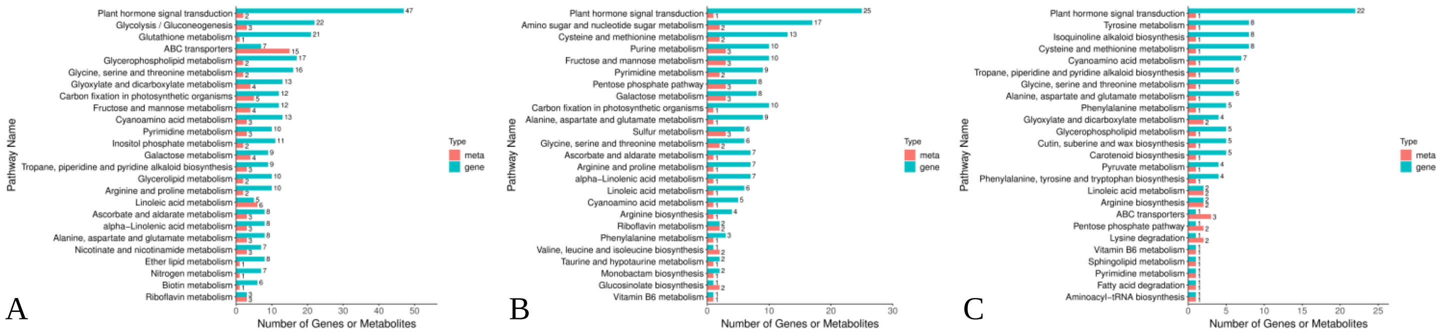

Figure S5. KEGG enrichment conjoint analyses of common DEGs and DAMs (A: ML vs. RL; B: YL vs. RL; C: ML vs. YL).

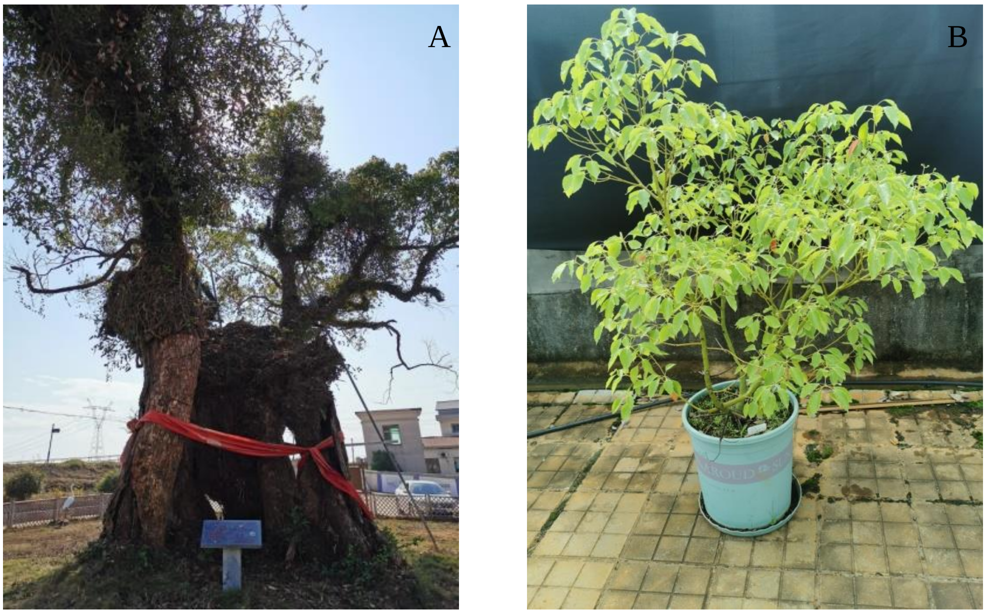

Figure S6. The 2000-year-old ancient *C. camphora* and its 2-year-old cutting seeding (A: 2000-year-old ancient *C. camphora*; B: 2-year-old cutting seeding)
